# The Effect of Vitamin A on Fracture Risk: A Meta-Analysis of Cohort Studies

**DOI:** 10.3390/ijerph14091043

**Published:** 2017-09-10

**Authors:** Xinge Zhang, Rui Zhang, Justin B. Moore, Yueqiao Wang, Hanyi Yan, Yingru Wu, Anran Tan, Jialin Fu, Ziqiong Shen, Guiyu Qin, Rui Li, Guoxun Chen

**Affiliations:** 1 School of Health Sciences, Wuhan University, 115 Donghu Road, Wuhan 430071, China; ylcz2920@126.com (X.Z.); elle529@126.com (Y.W.); yanlq@hotmail.com (H.Y.); yingru.wu@hotmail.com (Y.W.); chloetar@hotmail.com (A.T.); Fjl708326@163.com (J.F.); zq_shen666@163.com (Z.S.); yuu_qin@163.com (G.Q.); 2 College of Life Sciences, South-Central University for Nationalities, Wuhan 430074, China; zhangrui@mail.scuec.edu.cn; 3 Department of Family and Community Medicine, Wake Forest School of Medicine, Medical Center Boulevard, Winston-Salem, NC 27157, USA; jusmoore@wakehealth.edu; 4 Department of Epidemiology and Prevention, Wake Forest School of Medicine, Medical Center Boulevard, Winston-Salem, NC 27157, USA; 5 Department of Nutrition, The University of Tennessee, Knoxville, TN 37996, USA; gchen6@utk.edu

**Keywords:** vitamin A, retinol, β-carotene, hip fracture, total fracture

## Abstract

This meta-analysis evaluated the influence of dietary intake and blood level of vitamin A (total vitamin A, retinol or β-carotene) on total and hip fracture risk. Cohort studies published before July 2017 were selected through English-language literature searches in several databases. Relative risk (RR) with corresponding 95% confidence interval (CI) was used to evaluate the risk. Heterogeneity was checked by Chi-square and I^2^ test. Sensitivity analysis and publication bias were also performed. For the association between retinol intake and total fracture risk, we performed subgroup analysis by sex, region, case ascertainment, education level, age at menopause and vitamin D intake. R software was used to complete all statistical analyses. A total of 319,077 participants over the age of 20 years were included. Higher dietary intake of retinol and total vitamin A may slightly decrease total fracture risk (RR with 95% CI: 0.95 (0.91, 1.00) and 0.94 (0.88, 0.99), respectively), and increase hip fracture risk (RR with 95% CI: 1.40 (1.02, 1.91) and 1.29 (1.06, 1.57), respectively). Lower blood level of retinol may slightly increase total fracture risk (RR with 95% CI: 1.11 (0.94, 1.30)) and hip fracture risk (RR with 95% CI: 1.27 (1.05, 1.53)). In addition, higher β-carotene intake was weakly associated with the increased risk of total fracture (RR with 95% CI: 1.07 (0.97, 1.17)). Our data suggest that vitamin A intake and level may differentially influence the risks of total and hip fractures. Clinical trials are warranted to confirm these results and assess the clinical applicability.

## 1. Introduction

Osteoporosis, defined as a bone mineral density 2.5 standard deviations (SDs) or more below the average value of young healthy women (a *T*-score of <–2.5 SD) [1], is one of the major health issues worldwide. It is a skeletal disorder characterized by a low bone mass and micro architectural deterioration of the bone tissue with increased susceptibility to fracture [2], the common sites of which include the spine, hip, forearm and proximal humerus. Osteoporosis and its related fractures have a serious influence on the health, quality of life, and independence in the older adult population. The prevention and management on osteoporosis and its related fractures represent a serious burden on society and health care agencies [3]. Among the potential complications of osteoporosis, hip fracture is the most likely to be fatal [4,5]. In 2005, direct osteoporotic fractures cost the U.S. approximately $17 billion, with 72% of the cost attributable to hip fractures, although only 14% of incident fractures were of the hip [6]. The annual worldwide direct and indirect costs of hip fractures had been estimated to be $131 billion by 2050 [7]. In addition, 158 million individuals aged 50 years and up were at high risk of osteoporotic fracture worldwide in 2010, and this number was estimated to double by 2040 [8].

Vitamin A (VA) is a generic term for compounds with the biological activity of retinol [9]. Preformed VA (mainly retinol and retinyl esters) is usually found in foods derived from animal products, and provitamin A (mainly β-carotene and carotenoids) is absorbed when eating foods derived from plant products [9]. As a micronutrient, VA has been recognized as an important modifiable factor in the development and maintenance of bone mass. In 1934, Davies et al. observed spontaneous leg fracture after giving massive VA distillate (12–14 mg/day) to rats [10]. Since then, several other animal studies had also shown that excessive VA intake may cause toxicity on bone [11] through decreasing bone formation [12], increasing bone resorption [13], and enhancing fracture probability [14]. In addition, a number of population-based cohort studies demonstrated that higher dietary VA intake or its blood level might increase the risk of fracture (particularly hip fracture) [15,16]. However, the association between VA level and fracture risk at the population level has been controversial. It had been reported that neither high amount of VA intake nor serum VA level is strongly correlated with osteoporosis and fracture [17,18,19,20]. In addition, Jonge et al. found that higher VA intake might decrease fracture risk [21]. Similarly, a cohort study conducted by Barker et al. demonstrated that higher blood retinol level or blood total VA level was associated with a significant lower risk of total fracture [22]. As a precursor to VA, β-carotene has been indicated in the maintenance of bone health. The protective role of β-carotene for bone health was observed in both experimental and population-based studies [23,24,25]. However, several other studies suggested that β-carotene was not strongly correlated with fracture risk [20,26] and that β-carotene may increase fracture risk [22]. Thus, the present study aimed to evaluate the effects of VA intake and its blood level on fracture by conducting a meta-analysis of published cohort studies.

## 2. Materials and Methods

Our meta-analysis was performed in accordance with the Preferred Reporting Items for Systematic Reviews and Meta-Analyses (PRISMA) statement [27].

### 2.1. Search Strategy

A wide range of original research and observational studies published before January 2017, examining the association between VA and osteoporosis and its related fracture, were searched within the following databases: Medline, Embase, Science Online, Science Direct, JAMA and Nature. Combinations of at least two of the following key words: “osteoporosis”, “fracture”, “bone density”, “bone health”, “bone rarefaction” and “VA”, “retinol”, “β-carotene”, were used as search terms in the title/abstract of articles. Articles that met the inclusion criteria were retrieved for full-text review, unless the article was not available after all attempts to retrieve. In addition, we manually searched relevant publications by screening the bibliography of selected articles (published during 2014–2016), relevant reviews, and meta-analyses. These articles were not included unless original data were found. To reduce the potential selection bias, each article was independently evaluated by two of the investigators (Jialin Fu and Ziqiong Shen), and a mutual decision was made jointly in regard to whether or not it met the basic inclusion criteria. The disagreements were solved by the opinion of the third investigator (Xinge Zhang), and consensus was reached after discussion.

### 2.2. Inclusion Criteria

The inclusion criteria were listed as follows: (1) subjects were limited to adults; (2) original and cohort studies published in English-language refereed journals; (3) the exposure of interest was dietary or circulating VA (total VA, retinol or β-carotene); (4) the outcome was risk of total or hip fracture; (5) estimation of odds ratio (OR), RR or hazard ratio (HR) with 95% CI was provided.

### 2.3. Data Extraction

Data extraction was conducted using standard data extraction form by one investigator (Guiyu Qin) and checked by another one (Xinge Zhang). Extracted information included: title, last name of the first author, country, follow-up period, number and age of subjects, measure or range of exposure, research endpoint, case ascertainment, HR/OR/RR with 95% CI, variables adjusted for analysis.

### 2.4. Quality Assessment

We used the Newcastle-Ottawa Scale (NOS) to assess the quality of included studies [28]. The specific items were as follows: (1) representativeness of the exposed cohort; (2) selection of the non-exposed cohort; (3) ascertainment of exposure; (4) demonstration that outcome of interest was not present at start of study; (5) comparability of cohorts on the basis of the design or analysis; (6) assessment of outcome; (7) follow-up long enough for outcomes to occur; (8) adequacy of follow up of cohorts. For the fifth item, a maximum of two stars can be given. For the other items, a maximum of one star was assigned when the study was in consistent with the answer. The study was defined as having a high quality if the total stars were no less than 6.

### 2.5. Statistical Analysis

The measures of effects were RR, OR and HR with their corresponding 95% CIs. We treated the HR as RR and we converted OR into RR according to the method by Zhang et al. [29]. Some studies presented results using figures, instead of reporting specific estimate values with corresponding CI, so the software GetData Graph Digitizer 2.26 (http://getdata-graph-digitizer.com/) was applied to digitize and extract the data. Standard errors were calculated from 95% CIs with a formula [30]. Some studies reported the estimate values under different multivariate models, and we extracted the most stringently controlled estimate.

For studies researching VA (total VA, retinol or β-carotene) intake and risk of total or hip fracture, we extracted, pooled and synthesized values and corresponding 95% CI of the highest versus the lowest category of retinol intake. Subgroup analyses stratified by sex, region, case ascertainment, education level, age at menopause and vitamin D intake were conducted for meta-analysis of retinol intake and total fracture. The random effects model [31] was used for synthesis with high heterogeneity and the fixed effects model [32] was used for others.

For blood level of VA (total VA, retinol or β-carotene) and risk of total or hip fracture, we extracted estimate values with their corresponding 95% CIs of both the highest and lowest values versus middle category. There were too few studies concerning β-carotene, total VA and fracture to carry out a meta-analysis, we therefore only analyzed the relationship between blood retinol and total or hip fracture.

The statistical heterogeneity was assessed by Chi-square and I^2^ test [33]. *p* value < 0.1 and I^2^ > 50% indicated high heterogeneity. For sensitivity analysis, we omitted one study each time and then calculated the pooled RR and 95% CI to identify the studies that included potential confounding factors. Funnel plots and Egger’s linear regression method were used to evaluate potential publication bias [34]. All statistical analyses were performed through R software (R Foundation for Statistical Computing, Vienna, Austria).

## 3. Results

### 3.1. Literature Search

**Figure 1:**
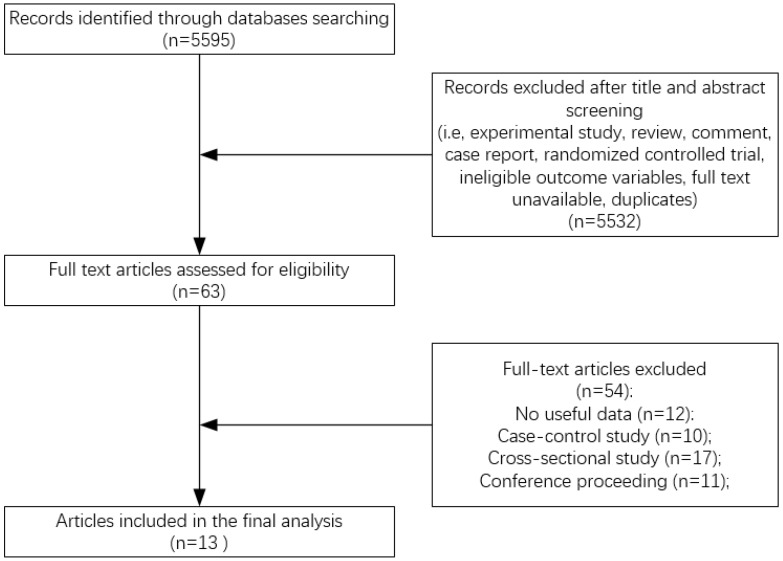
Flow diagram of literature search.

After the title and abstract screening followed by the full-text screening, a total of 13 articles (11 prospective cohort studies and 2 nested case-control studies) published between 2002 and 2015 were included based on our inclusion criteria. Figure 1 presented our search and selection process. Out of these articles, one study provided odds ratio (OR) with 95% confidence interval (CI) [35], four provided relative risks (RRs) with 95% CIs [17,20,21,26] and eight provided hazard ratios (HRs) with 95% CIs [16,18,19,22,25,36,37,38]. We considered a study that separately presented results for men and women [20,25,37] as two independent studies. In addition, two studies presented results based on VA intake from food-only and food plus supplement [19,21]. We extracted the “food plus supplement” data for our analyses. In total, there were eight studies investigating the association between VA (total VA, retinol or β-carotene) intake and fracture risk (hip or total) [16,19,20,21,25,26,35,36] and five studies investigating the relationship between blood level of retinol and fracture risk (hip or total) [17,18,22,37,38].

### 3.2. Study Characteristics

Characteristics of eight studies investigating the relationship between VA (total VA, retinol or β-carotene) intake and fracture risk (hip or total) [16,19,20,21,25,26,35,36] are summarized in Table 1. A total of 307,093 participants (109,056 post-menopausal women) over the age of 20 years were included in our meta-analysis. 21,448 cases of total fracture and 3593 cases of hip fracture were identified. One article did not provide fracture cases [16]. Three studies were carried out in America [19,21,22] and the rest were in British [20], Denmark [36], Netherlands [16], Sweden [35] and Singapore [25] with one study in each country. VA (retinol, β-carotene and total VA) consumption was estimated by self-reported questionnaire and interview. Follow-up time ranged from 3–18 years. One article did not provide retinol and β-carotene intake values [16].

Characteristics of five studies regarding the relationship between blood retinol level and risk of fracture (hip or total) are summarized in Table 2 [17,18,22,37,38]. A total of 11,984 subjects over the age of 39 years were included in our meta-analysis. 2219 cases (1602 hip fracture and 701 total fracture) were identified with a follow-up time ranging from 6 to 31 years. These studies were carried out in Sweden [17], Australia [18], Norway [37], America [38] and British [22] with one study in each country. Serum level of retinol was measured by high-performance liquid chromatography.

### 3.3. Total VA Intake and Risk of Fracture

**Figure 2:**
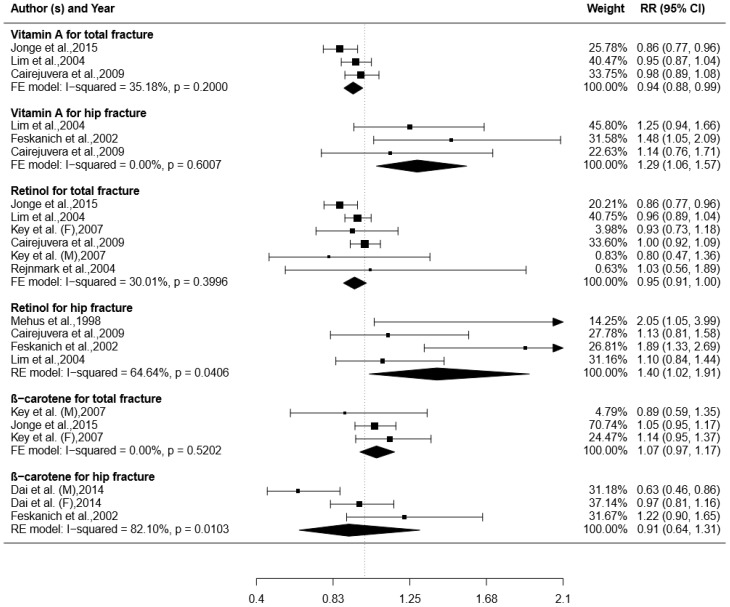
Association between VA (total VA or retinol or β-carotene) intake and risk of total or hip fracture. Boxes represent RR for each individual study; horizontal lines represent 95% CI; arrows indicate CI larger than 2.1; rhombus represent the combined RR with 95% CI.

Three studies were included for the analyses of the relationship between total VA intake and total fracture [16,19,21] while three were included for the analysis of the relationship between the total VA intake and hip fracture [19,21,26]. As shown in Figure 2, the higher total VA intake may be associated with the decrease of the risk of total fracture (RR with 95% CI: 0.94 (0.88, 0.99)), and the increase of the risk of hip fracture (RR with 95% CI: 1.29 (1.06, 1.57)). There was no significant heterogeneity in the analyses of the relationship of between the total VA intake and total fracture (I^2^ = 35.18%, *p* = 0.20) or hip fracture (I^2^ = 0.00%, *p* = 0.60).

### 3.4. Retinol Intake and Risk of Fracture

Five and four studies respectively reported the relationship between retinol intake and total fracture and the relationship between retinol intake and hip fracture were included [19,21,26,35]. Pooled results showed that the increased retinol intake might decrease the risk of total fracture (RR with 95% CI: 0.95 (0.91, 1.00)) and increase the risk of hip fracture (RR with 95% CI: 1.40 (1.02, 1.91)). As shown in Figure 2, significant heterogeneity was observed in the analyses of the relationship between retinol and hip fracture (I^2^ = 64.64%, *p* = 0.04), but not in that between retinol and total fracture (I^2^ = 30.01%, *p* = 0.40).

### 3.5. β-carotene Intake and Risk of Fracture

Two studies were included to analyze the relationship between β-carotene intake and total fracture [16,20], and two more were for the relationship between β-carotene intake and hip fracture [25,26]. We found that higher β-carotene intake is slightly associated with the increase of the risk of total fracture (RR with 95% CI: 1.07 (0.97, 1.17)), and with a tendency to decrease the risk of hip fracture (RR with 95% CI: 0.91 (0.64, 1.31)). Significant heterogeneity was observed in the analyses of the relationship between β-carotene intake and total fracture (I^2^ = 0.00%, *p* = 0.52), but not that between β-carotene intake and hip fracture (I^2^ = 82.10%, *p* = 0.01) (Figure 2).

### 3.6. Blood Level of Retinol and Risk of Fracture

**Figure 3:**
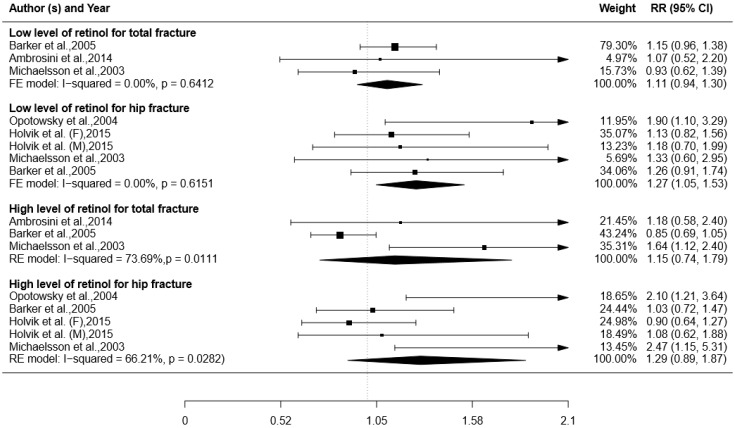
Association between blood level of retinol and risk of total or hip fracture. Boxes represent RR for each individual study; horizontal lines represent 95% CI; arrows indicate CI larger than 2.1; rhombus represent the combined RR with 95% CI.

Five studies were pooled for the analyses of association between blood level of retinol and fracture (total or hip) [17,18,22,37,38]. We observed slightly positive impact of low blood level of retinol on fracture (RR with 95% CI: total fracture = 1.11 (0.94, 1.30); and hip fracture = 1.27 (1.05, 1.53)). There was no significant heterogeneity in studies on low blood level of retinol for total fracture (I^2^ = 0.00%, *p* = 0.64) or for hip fracture (I^2^ = 0.00%, *p* = 0.62) (Figure 3).

### 3.7. Subgroup Analysis, Sensitivity Analysis, and Publication Bias

Subgroup analyses were performed to determine the relationship between retinol intake and total fracture risk, and the results were shown in Table 3. Subgroups of men (RR with 95% CI: 0.80 (0.47, 1.36)) and mixed gender (RR with 95% CI: 0.86 (0.77, 0.96)) had lower total fracture risks than the subgroup of women (RR with 95% CI: 0.98 (0.92, 1.03)). Subjects in European had lower total fracture risk (RR with 95% CI: 0.87 (0.79, 0.96)) than those in America (RR with 95% CI: 0.98 (0.92, 1.03)). Different total fracture ascertainments showed different results (RR with 95% CI: self-report = 0.97 (0.92, 1.03); medical record = 0.87 (0.78, 0.96)). In addition, pooled results of studies adjusted for age at menopause showed a lower total fracture risk than those unadjusted (RR with 95% CI: 0.87 (0.78, 0.96)) did. Subgroup analyses suggested that sex, region, case ascertainment, and age at menopause were potential factors.

By omitting one study each time using a random effects model [31], sensitivity to each study was found in the analysis of the relationships between β-carotene intake and hip fracture, between high blood level of retinol and total fracture, and between low blood level of retinol and total fracture. As shown in Table 4, inverse results were obtained after omitting the studies conducted by Feskanich et al. [26], Dai et al. (Female) [25], Michaelsson et al. (Total) [17] and Barker et al. [22]. Egger’s test revealed the presence of publication bias only in the analysis of the relationship between high blood level of retinol and hip fracture (*p* = 0.005) (Table 4).

## 4. Discussion

### 4.1. Higher Total VA or Retinol Intake may Decrease Total Fracture Risk but Increases Hip Fracture Risk

Our results suggested that higher intake of total VA or retinol slightly increased the risk of hip fracture. A number of cohort studies indicated a similar trend (not statistically significant) [19,21], highlighting the benefit of this meta-analysis. It has been shown that the excessive retinol intake can result in bone lesions, calcification of organs [39], stimulation of osteoclast formation [13] and suppression of osteoblast activity [12].

In addition, the interactions between retinol and vitamin D or calcium may play a role. Excessive retinol intake can alter the metabolism of calcium-regulating hormones [40] and eliminate the ability of vitamin D to maintain the level of serum calcium [14]. In contrast, we found a negative influence of higher level of total VA or retinol intake on total fracture risk, which is consistent with the study conducted by Jonge et al. [16]. On the other hand, several studies have shown that higher retinol intake may increase bone mineral density [41,42]. This discrepancy can be partially explained by the fact that the relationship between retinol intake and bone mineral density varies by bone site [43,44,45]. Houtkooper et al. [44] measured bone mineral density at lumbar vertebrae 2–4, femur neck, Ward’s triangle, and Trochanter. They found a positive association between retinol intake and bone mineral density at all bone sites except for femoral neck. Given the fact that the hip is as sensitive to retinol intake as femoral neck [46], it is reasonable to assume that higher VA or retinol intake may result in differential fracture risks depending on bone sites analyzed.

Our findings are not consistent with the results reported in a previous meta-analysis conducted by Wu et al. [47]. The authors concluded that high intake of VA or retinol might increase the risk of hip fracture, but not that of total fracture. This could be attributed to the study [16] that was included in the current analysis, but not in that by Wu et al. [47].

### 4.2. Subgroup Analysis for Retinol Intake and Total Fracture Risk

Subgroup analysis for retinol intake and total fracture suggested that sex, age at menopause, case ascertainment and region might be potential factors, which were not reported in a previous meta-analysis by Wu et al. [47]. It has been shown that, compared with men, women have lower bone strength, bone mineral content and bone density [48,49], and therefore face higher fracture risk. Sioka et al. reported that age of women between 40 and 45 years old at menopause was correlated with low bone mineral density in comparison with that of postmenopausal women [50]. In addition, a 9% disagreement between self-reported fracture and medical record was found in the study by Bush et al. [51], which may contribute to the variations. Moreover, dietary retinol intake in European subjects was lower than that in U.S. subjects, which may contribute to the variance of total fracture risk.

### 4.3. Higher β-Carotene Intake may Increase Total Fracture Risk

Here. A positive association between β-carotene intake and risk of total fracture was found. Interestingly, Wu et al. [47] reported that β-carotene intake was not associated with the risk of total fracture. This inconsistency could be explained by the fact that we included one article [16] published in 2015 that could not be analyzed by Wu et al. [47]. Previous studies on the relationship between β-carotene intake and fracture risk reported controversial results. Several population-based studies demonstrated protective effect of β-carotene on bone [52,53]. However, Wolf et al. [54] and Melhus et al. [55] have shown a negative influence of β-carotene on bone. As a provitamin, A.; β-carotene is mainly converted to retinal and then from retinal to retinol in the cytosol of intestinal mucosal cells. This is regulated in vivo by the levels of β-carotene and retinol [56]. High retinol intake suppresses the conversion of β-carotene to retinol [57]. Since we did not observe significant effect of blood retinol level on fracture risk, it seems that β-carotene itself may affect bone health. It has been shown that β-carotene suppresses osteoclast formation [58], and that β-carotene may promote osteoblast mineralization [59]. Therefore, the specific effect of β-carotene on bone was unclear and merited further investigation.

### 4.4. Lower Blood Level of Retinol may Increase Total and Hip Fracture Risk

Previous study reported a U-shaped relationship between serum retinol and hip fracture risk [38]. Consistently, our results suggest that lower blood level of retinol increases both total and hip fracture risk. On the other hand, the results of higher blood level of retinol did not reach statistical significance. It has been shown that VA deficiency may affect sulfation of proteoglycans [60], decrease the expression of growth hormone, insulin-like growth factor, and insulin-like growth factor–binding protein 3 [61], which in turn, may reduce the bone density and strength.

### 4.5. Limitations

This meta-analysis has several potential limitations. First, we failed to obtain the exposure categories and RR with 95% CI of our interests in the study conducted by Sahni et al. [24], which was excluded in the analysis. Second, we failed to observe the association between lower VA intake and fracture risk as all included studies took the lowest intake as reference except the study by Jonge et al. [16]. Third, sensitivity to individual study and publication bias were found. Fourth, different studies assessed dietary intake depending on different food frequency questionnaire, and the semi quantitative instrument might cause misclassification of dietary intake. Fifth, some included studies only collected baseline data, and there might be secular changes in dietary structure during the follow-up period. Sixth, several included studies assessed retinol level based on fasting blood while the others did not [62]. Seventh, some studies included heterogeneous subjects in terms of age [20,26], and in vivo retinol concentration may increase with age [63].

## 5. Conclusions

Our meta-analysis suggested that higher total VA or retinol intake might slightly decrease the risk of total fracture and increase the risk of hip fracture. In addition, we found that higher β-carotene intake increases the risk of total fracture slightly but not hip fracture. Moreover, we observed weak but positive influence of lower blood retinol level on both total and hip fracture risk.

## Figures and Tables

**Table 1:** Characteristics of studies on VA (total VA, retinol or β-carotene) intake.

Author Year [ref.]	Country Period	Male/Female Age Case	Range of Exposure	Exposure Assessment	Case Ascertainment	Adjusted Variables	Quality Score
Feskanich et al., 2002 [26]	U.S. 1980–1998	0/72,337 34–77 Hip: 603	Total VA μg RE/day	FFQ	Self-report	1, 2, 3, 4, 9, 10, 11, 12, 14, 15, 17, 18, 23	8
			<1250; 1250–1699; 1700–2249; 2250–2999; ≥3000				
			Retinol μg/day				
			<500; 500–849; 850–1299; 1300–1999; ≥2000				
			β-carotene μg/day				
			<2550; 2550–3549; 3550–4669; 4650–6299; ≥6300				
Melhus et al., 1998 [35]	SE 1987–1990	0/1120 40–76 Hip: 247	Retinol μg/day	FFQ	Medical records	2, 5, 8, 9, 11, 12, 16, 17, 20, 32	7
			≤500; 500–1000; 1000–1500; >1500				
Lim et al., 2004 [21]	US 1985–1997	0/34,703 55–69 Total: 6502 Hip: 525	Total VA IU	FFQ	Self-report	1, 2, 7, 8, 10, 17, 19, 20, 24	6
			221–7055; 7056–10484; 10,485–14,209; 14,210–19,892; 19,893–23,6991				
			Retinol IU				
			28–1405; 1406–2952; 2953–4655; 4656–7001; 7002–211,051				
Key et al., 2007 [20]	UK 1993–2005	7947/26,749 20–89 Total: 1898	Retinol μg/day	FFQ	Self-report	1, 2, 3, 4, 8, 9, 10, 11, 14, 22, 25, 26, 27, women for 21 and 12	8
			<200; 200–299; 300–499; 450–999; ≥1000				
			β-carotene μg/day				
			<2000; 2000–2999; 3000–3999; 4000–4499; ≥5000				
Dai et al., 2014 [25]	SG 1993–2010	27,959/35,298 45–74 Hip: 1630	β-carotene 1000 kcal/day	FFQ	Medical records	1, 2, 4, 7, 8, 9,11, 12, 13, 20, 29, 30, 31, 32	8
			<850.4; 580.4–1235.4; 1235.4–1772.4; ≥1772.4				
Cairejuvera et al., 2009 [19]	U.S. 1993–2005	0/93676 50–79 Total: 10,405 Hip: 588	Total VA μg RE/day	FFQ	Self-report for total; Medical records for hip	1, 2, 3, 4, 8, 9, 10, 11, 12, 14, 15, 18, 28, 31	8
			<5055; 5055–5824; 5825–6550; 6551–7507; ≥7508				
			Retinol μg/day				
			<474; 474–764; 765–1092; 1093–1425; ≥1426				
Rejnmark et al., 2004 [36]	DK 5 years	0/2016 45–58 Total: 163	Retinol μg/day	FFQ	Medical records	1, 3, 4, 5, 6, 8, 9, 10, 11, 16, 17, 33, 34, 35, 36	8
			<500; 500–1500; >1500				
Jonge et al., 2015 [16]	NED 1989–2007	2172/3116 ≥ 50	Total VA μg RE/day	FFQ	Medical records	1, 2, 4, 5, 7, 9, 10, 11, 12, 37, 38, 39	7
			568–793; 867–1052; 1050–1257; 1263–1551; 1712–2485				
			Retinol μg/day				
			135–289; 173–384; 212–523; 272–734; 594–1518				
			β-carotene μg/day				
			1845–2909; 2942–3964; 3336–4575; 3586–5461; 3771–6586				

VA: Vitamin A; RE: retinol equivalent (RE = μg retinol+(μg β-carotene/6) + (μg α-carotene/12) + (μg β-cryptoxanthin/24)); FFQ: food frequency questionnaire; Adjusted variables definition: 1: age; 2: BMI (body mass index); 3: vitamin D intake; 4: calcium intake; 5: age at: menopause; 6: vitamin D supplement intake; 7: education; 8: energy intake; 9: smoking; 10: alcohol intake; 11: physical activity; 12: hormone replacement therapy use; 13: year of recruitment; 14: protein intake; 15: vitamin K; 16: previous fracture; 17: medicine use; 18: caffeine intake; 19: cirrhosis; 20: diabetes mellitus; 21: parity; 22: marital status; 23: follow-up cycle; 24: past irregular menstrual duration; 25: vitamin C intake; 26: Potassium; 27: Magnesium; 28: region; 29: ethnic group; 30: soy isoflavones intake; 31: vitamin B6; 32: menopausal status: ; 33: weight; 34: bone mineral density; 35: VA intake; 36: β-carotene intake; 37: sex; 38: net income; 39: disability index.

**Table 2:** Characteristics of studies on blood level of retinol.

Author Year [ref.]	Country Period	Population/Age Case	Category of Blood Retinol (μmol/L)	Exposure Assessment	Case Ascertainment	Adjusted Variables	Quality Score
Michaelsson et al., 2003 [17]	SE 1970–2001	2322/49–51 Total: 266 Hip: 84	The lowest: <1.95 The highest: >2.64	High-performance liquid chromatography	Medical records	1, 2, 3, 4, 5, 6, 7, 8, 9, 10, 11, 12	9
Ambrosini et al., 2014 [18]	AU 1990–2007	998/39–62 Total: 123	The lowest: ≤2.80 The highest: ≥19.3	High-performance liquid chromatography	Self-reported	1, 8, 13,14, 15	6
Holvik et al., 2015 [37]	NO 1994–2008	2487/65–79 Hip: 1154	The lowest: ≤2.12 The highest: >3.63	High-performance liquid chromatography	Medical records	1, 2, 16, 17	8
Barker et al., 2005 [22]	UK 1996–2002	2606/≥75 Total: 312 Hip: 192	The lowest: ≤1.66 The highest: ≥2.42	High-performance liquid chromatography	Medical records	Total: 1, 18 Hip: 1, 3, 18	8
Opotowsky et al., 2004 [38]	U.S. 1971–1992	0/3571 50–74 Hip: 172	The lowest: ≤1.61 The highest: ≥2.56	-	Medical records	1, 3, 7, 11, 12, 14, 15, 19, 20	8

1: age; 2: BMI (body mass index); 3: weight; 4: height; 5: serum β-carotene; 6: serum calcium; 7: serum albumin; 8: smoking; 9: marital status; 10: socioeconomic class; 11: physical activity; 12: alcohol consumption; 13: sex; 14: medications; 15: previous fracture; 16: study center; 17: serum α-tocopherol; 18: total hip bone mineral density; 19: race; 20: dietary calcium intake.

**Table 3:** Subgroup analyses for retinol intake and total fracture.

Exposure	Subgroup	Number of Studies	RR (95% CI)	Q	*p*-Q	I^2^ (%)
Sex	Men	1	0.80 (0.47, 1.36)	0.00	1.00	-
	Women	4	0.98 (0.92, 1.03)	0.69	0.88	0.00%
	All	1	0.86 (0.77, 0.96)	0.00	1.00	-
Region	USA	2	0.98 (0.92, 1.03)	0.50	0.48	0.00%
	Europe	4	0.87 (0.79, 0.96)	0.70	0.87	0.00%
Case ascertainment	Self-report	4	0.97 (0.92, 1.03)	1.19	0.76	0.00%
	Medical record	2	0.87 (0.78, 0.96)	0.32	0.57	0.00%
Education level	Adjusted	2	0.93 (0.90, 0.97)	2.55	0.11	60.83%
	Unadjusted	4	0.99 (0.91, 1.07)	0.94	0.82	0.00%
Age at menopause	Adjusted	2	0.87 (0.78, 0.96)	0.32	0.57	0.00%
	Unadjusted	4	0.97 (0.92, 1.03)	1.19	0.76	0.00%
Vitamin D intake	Adjusted	3	0.95 (0.87, 1.02)	4.63	0.099	58.31%
	Unadjusted	3	0.92 (0.75, 1.13)	0.40	0.82	0.00%

**Table 4:** Sensitivity analyses and publication bias.

Group	Omitted Study	RR (95% CI)	Q	*p*-Q	I^2^ (%)	Z-Egger	*p*-Egger
Total VA intake for total fracture	Cairejuvera et al., [19]	0.93 (0.86, 1.00)	3.03	0.08	67.01%	−1.3982	0.16
	Lim et al., [21]	0.96 (0.90, 1.03)	0.22	0.64	0.00%		
	Feskanich et al., [26]	0.91 (0.85, 0.98)	1.89	0.17	47.11%		
Total VA for hip fracture	Jonge et al., [16]	1.34 (1.08, 1.67)	0.55	0.46	0.00%	−0.1893	0.85
	Cairejuvera et al., [19]	1.21 (0.96, 1.53)	0.13	0.72	0.00%		
	Lim et al., [21]	1.33 (1.02, 1.72)	0.93	0.34	0.00%		
Retinol intake for total fracture	Jonge et al., [16]	0.95 (0.90, 1.00)	5.07	0.28	36.04%	0.6236	0.53
	Cairejuvera et al., [19]	0.95 (0.91, 1.00)	5.10	0.28	39.45%		
	Key et al. (M), [20]	0.95 (0.91, 1.00)	4.73	0.32	34.86%		
	Key et al. (F), [20]	0.93 (0.87, 0.98)	2.96	0.56	20.26%		
	Lim et al., [21]	0.97 (0.92, 1.03)	1.22	0.87	0.00%		
	Rejnmark et al., [36]	0.94 (0.89, 1.00)	5.02	0.29	37.52%		
Retinol intake for hip fracture	Cairejuvera et al., [19]	1.54 (1.02, 2.33)	7.13	0.028	69.57%	1.3639	0.17
	Lim et al., [21]	1.57 (1.06, 2.31)	5.29	0.07	60.93%		
	Feskanich et al., [26]	1.17 (0.96, 1.43)	2.96	0.23	0.01%		
	Melhus et al., [35]	1.31 (0.94, 1.84)	6.48	0.039	70.25%		
β-carotene intake for total fracture	Jonge et al., [16]	1.04 (0.94, 1.16)	0.61	0.44	0.00%	−0.4368	0.66
	Key et al. (F), [20]	1.08 (0.98, 1.18)	0.54	0.46	0.00%		
	Key et al. (M), [20]	1.09 (0.93, 1.29)	1.16	0.28	14.10%		
β-carotene intake for hip fracture	Dai et al. (M), [25]	0.88 (0.46, 1.68)	8.76	0.03	88.59%	−0.2493	0.80
	Dai et al. (F), [25]	1.05 (0.85, 1.30)	1.62	0.20	38.10%		
	Feskanich et al., [26]	0.80 (0.52, 1.22)	5.45	0.02	81.64%		
High level of blood retinol for total fracture	Michaelsson et al., [17]	0.87 (0.71, 1.07)	0.75	0.39	0.00%	0.4619	0.64
	Ambrosini et al., [18]	1.16 (0.61, 2.20)	8.78	0.003	88.61%		
	Barker et al., [22]	1.52 (1.09, 2.13)	0.64	0.42	0.00%		
High level of blood retinol for hip fracture	Michaelsson et al., [17]	1.15 (0.82, 1.61)	6.78	0.079	57.69%	2.7952	0.005
	Barker et al., [22]	1.41 (0.87, 2.29)	10.27	0.016	70.12%		
	Holvik et al. (M), [37]	1.37 (0.85, 2.21)	10.79	0.013	76.05%		
	Holvik et al. (F), [37]	1.46 (0.95, 2.24)	7.67	0.053	61.11%		
	Opotowsky et al., [38]	1.08 (0.84, 1.40)	5.62	0.13	23.13%		
Low level of blood retinol for total fracture	Michaelsson et al., [17]	1.15 (0.96, 1.36)	0.04	0.85	0.00%	−0.6384	0.52
	Ambrosini et al., [18]	1.11 (0.94, 1.31)	0.88	0.35	0.00%		
	Barker et al., [22]	0.96 (0.68, 1.37)	0.11	0.74	0.00%		
Low level of blood retinol for hip fracture	Michaelsson et al., [17]	1.26 (1.04, 1.54)	2.65	0.45	0.00%	0.8156	0.41
	Barker et al., [22]	1.27 (1.01, 1.60)	2.66	0.45	0.00%		
	Holvik et al. (F), [37]	1.35 (1.06, 1.71)	1.92	0.59	0.00%		
	Holvik et al. (M), [37]	1.28 (1.04, 1.57)	2.58	0.46	0.00%		
	Opotowsky et al., [38]	1.20 (0.98, 1.47)	0.29	0.96	0.00%		

## Abbreviations

CI	Confidence interval
FFQ	Food frequency questionnaire
HR	Hazard risk
NOS	Newcastle-Ottawa Scale
OR	Odds ratio
PSRMA	Preferred Reporting Items for Systematic Reviews and Meta-Analyses
RR	Relative risk
VA	Vitamin A
